# Advisory Committee on Immunization Practices Recommended Immunization Schedule for Adults Aged 19 Years or Older — United States, 2017

**DOI:** 10.15585/mmwr.mm6605e2

**Published:** 2017-02-10

**Authors:** David K. Kim, Laura E. Riley, Kathleen H. Harriman, Paul Hunter, Carolyn B. Bridges

**Affiliations:** ^1^Immunization Services Division, National Center for Immunization and Respiratory Diseases, CDC; ^2^Harvard University; ^3^California Department of Public Health; ^4^University of Wisconsin.

In October 2016, the Advisory Committee on Immunization Practices (ACIP) voted to approve the Recommended Adult Immunization Schedule for Adults Aged 19 Years or Older—United States, 2017. The 2017 adult immunization schedule summarizes ACIP recommendations in two figures, footnotes for the figures, and a table of contraindications and precautions for vaccines recommended for adults. These documents are available at https://www.cdc.gov/vaccines/schedules. The full ACIP recommendations for each vaccine can be found at https://www.cdc.gov/vaccines/hcp/acip-recs/index.html. The 2017 adult immunization schedule was also reviewed and approved by the American College of Physicians (https://www.acponline.org), the American Academy of Family Physicians (https://www.aafp.org), the American College of Obstetricians and Gynecologists (http://www.acog.org), and the American College of Nurse-Midwives (http://www.midwife.org).

A cover page has been added to the 2017 adult immunization schedule that contains information on select general principles pertinent to the adult immunization schedule, additional CDC resources, instructions for reporting vaccine adverse events related to vaccination and suspected cases of reportable vaccine-preventable diseases, and an ACIP-approved list of standardized acronyms for vaccines recommended for adults.* In addition, the table of contraindications and precautions for vaccines routinely recommended for adults, which was formerly a stand-alone document, has been incorporated into the adult immunization schedule.

## Changes in the 2017 Adult Immunization Schedule

Changes in the 2017 adult immunization schedule from the previous year’s schedule include new or revised ACIP recommendations for influenza, human papillomavirus, hepatitis B, and meningococcal vaccines:

**Influenza vaccination (*****1*****).** Changes are related to the low effectiveness of the live attenuated influenza vaccine (LAIV) (FluMist, MedImmune) against influenza A(H1N1)pdm09 in the United States during the 2013–2014 and 2015–2016 influenza seasons and revised recommendations for the use of influenza vaccine among patients with egg allergy. These changes are reflected in the 2017 adult immunization schedule as follows:

- LAIV should not be used during the 2016–2017 influenza season.
- Adults with a history of egg allergy who have only hives after exposure to egg should receive age-appropriate inactivated influenza vaccine (IIV) or recombinant influenza vaccine (RIV).
- Adults with a history of egg allergy with symptoms other than hives (e.g., angioedema, respiratory distress, lightheadedness, or recurrent emesis, or who required epinephrine or another emergency medical intervention) may receive age-appropriate IIV or RIV. The selected vaccine should be administered in an inpatient or outpatient medical setting and supervised by a health care provider who is able to recognize and manage severe allergic conditions.

**Human papillomavirus vaccination (*****2*****).** Healthy adolescents who start their human papillomavirus (HPV) vaccination series before age 15 years are recommended to receive 2 doses of HPV vaccine. Adults and adolescents who did not start their HPV vaccination series before age 15 years should receive 3 doses of HPV vaccine. Changes in recommendations in the adult immunization schedule include updates regarding HPV vaccination for adults who did not complete the HPV vaccination series as adolescents. These changes are described in the 2017 adult immunization schedule as follows:

- Adult females through age 26 years and adult males through age 21 years who have not received any HPV vaccine should receive a 3-dose series of HPV vaccine at 0, 1–2, and 6 months. Males aged 22 through 26 years may be vaccinated with a 3-dose series of HPV vaccine at 0, 1–2, and 6 months.
- Adult females through age 26 years and adult males through age 21 years (and males aged 22 through 26 years who may receive HPV vaccine) who initiated the HPV vaccination series before age 15 years and received 2 doses at least 5 months apart are considered adequately vaccinated and do not need an additional dose of HPV vaccine.
- Adult females through age 26 years and adult males through age 21 years (and males aged 22 through 26 years who may receive HPV vaccine) who initiated the HPV vaccination series before age 15 years and received only 1 dose, or 2 doses less than 5 months apart, are not considered adequately vaccinated and should receive 1 additional dose of HPV vaccine.

**Hepatitis B vaccination (*****3*****). **The ACIP updated chronic liver disease conditions for which a hepatitis B vaccine (HepB) series is recommended. This change is described in the 2017 adult immunization schedule as follows:

- Adults with chronic liver disease, including, but not limited to, hepatitis C virus infection, cirrhosis, fatty liver disease, alcoholic liver disease, autoimmune hepatitis, and an alanine aminotransferase (ALT) or aspartate aminotransferase (AST) level greater than twice the upper limit of normal should receive a HepB series.

**Meningococcal vaccination (*****4*****,*****5*****).** There are two changes in meningococcal vaccination recommendations for 2017. First, the ACIP recommended that adults with human immunodeficiency virus (HIV) infection receive a 2-dose primary series of serogroups A, C, W, and Y meningococcal conjugate vaccine (MenACWY). Second, the ACIP provided updated dosing guidance for one of the serogroup B meningococcal vaccines (MenB) (MenB-FHbp [Trumenba, Pfizer]). Three doses of MenB-FHbp should be administered at 0, 1–2, and 6 months to adults who are at increased risk for meningococcal disease, and those who are vaccinated during serogroup B meningococcal disease outbreaks. When MenB-FHbp is given to healthy adolescents and young adults who are not at increased risk for meningococcal disease, 2 doses of MenB-FHbp should be administered at 0 and 6 months (MenB-FHbp was previously recommended as a 3-dose series at 0, 2, and 6 months, consistent with the original vaccine licensure for this population). The dosing frequency and interval for the other MenB, MenB-4C (Bexsero, GlaxoSmithKline), have not changed: MenB-4C remains a 2-dose series, with doses administered at least 1 month apart. Either MenB vaccine can be used when vaccination is indicated. The change in ACIP recommendations on the use of MenB-FHbp does not imply a preference for one MenB over the other. These updates in meningococcal vaccination are reflected in the 2017 adult immunization schedule as follows:

- Adults with anatomical or functional asplenia or persistent complement component deficiencies should receive a 2-dose primary series of MenACWY, with doses administered at least 2 months apart, and be revaccinated every 5 years. They should also receive a series of MenB with either MenB-4C (2 doses administered at least 1 month apart) or MenB-FHbp (3 doses administered at 0, 1–2, and 6 months).
- Adults with HIV infection who have not been previously vaccinated should receive a 2-dose primary MenACWY vaccination series, with doses administered at least 2 months apart, and be revaccinated every 5 years. Those who previously received 1 dose of MenACWY should receive a second dose at least 2 months after the first dose. MenB is not routinely recommended for adults with HIV infection, because meningococcal disease in this population is caused primarily by serogroups C, W, and Y.
- Microbiologists who are routinely exposed to isolates of *Neisseria meningitidis* should receive 1 dose of MenACWY and be revaccinated every 5 years if the risk for infection remains, as well as either MenB-4C (2 doses administered at least 1 month apart) or MenB-FHbp (3 doses administered at 0, 1–2, and 6 months).
- Adults at risk because of a meningococcal disease outbreak should receive 1 dose of MenACWY if the outbreak is attributable to serogroup A, C, W, or Y; or, if the outbreak is attributable to serogroup B, either MenB-4C (2 doses administered at least 1 month apart) or MenB-FHbp (3 doses administered at 0, 1–2, and 6 months).
- Young adults aged 16 through 23 years (preferred age range is 16 through 18 years) who are healthy and not at increased risk for serogroup B meningococcal disease may receive either MenB-4C (2 doses administered at least 1 month apart) or MenB-FHbp (3 doses administered at 0, 1–2, and 6 months) for short-term protection against most strains of serogroup B meningococcal disease.

**Notable changes to Figures 1 and 2.** Changes in “Figure 1. Recommended immunization schedule for adults aged 19 years or older, by age group” and “Figure 2. Recommended immunization schedule for adults aged 19 years or older by medical condition and other indications” are as follows:

- In Figures 1 and 2, standardized acronyms for vaccines are used to promote simplicity and consistency, and their listing has been reordered. Ancillary information previously contained in the figures has been consolidated and moved to the cover page. Colored blocks instead of colored bars are used to denote indications. These figures must be used in conjunction with the footnotes, which contain important information for each vaccine and considerations for special populations.
- In Figure 2, the columns for medical conditions and other indications have been reordered to keep medical conditions together and special populations together. Additional footnotes mark appropriate columns of medical conditions and other indications to refer the reader to view relevant vaccine-specific information.
- In Figure 2, the color of the indication block for MenACWY for HIV infection has been changed to yellow (recommended for adults who meet the age requirement, lack documentation of vaccination, or lack evidence of past infection) from purple (recommended for adults with additional medical conditions or other indications).

**Changes to footnotes.**

- Footnotes are limited to the information pertaining to vaccines listed in Figures 1 and 2 and are organized by vaccine-specific information and considerations for special populations (e.g., pregnant women and adults with HIV infection). The footnote labeled “additional information,” contained in previous versions of the adult immunization schedule, has been moved to the cover page. The footnote related to immunocompromising conditions has been removed, but vaccine-specific information on immunocompromising conditions has been added to the appropriate footnotes (e.g., the footnote for pneumococcal vaccination).
- The format for the footnotes has been condensed, simplified, and standardized. The format for pneumococcal; human papillomavirus; meningococcal; varicella; and measles, mumps, and rubella vaccination footnotes has undergone substantial revision.

**Other changes**. Lastly, the table of contraindications and precautions for vaccines routinely recommended for adults, which previously was a stand-alone document, has been incorporated into the adult immunization schedule. The content of the table has been consolidated and simplified.

## More Information

Details on these updates and information on other vaccines recommended for adults are available online under Adult Immunization Schedule, United States, 2017 (https://www.cdc.gov/vaccines/schedules/hcp/adult.html) and in the Annals of Internal Medicine (*6*). The full ACIP recommendations for each vaccine are also available online (https://www.cdc.gov/vaccines/hcp/acip-recs/index.html).

## Footnotes

* CDC encourages organizations that previously have relied on copying the adult immunization schedule on their websites to use syndication instead, as a more reliable method for displaying the most current and accurate adult immunization schedule. Use of content syndication requires a one-time step that ensures an organization's website displays the adult immunization schedule as soon as it is published or revised. The syndication code for the adult immunization schedule and instructions for its use can be found at https://www.cdc.gov/vaccines/schedules/syndicate.html. Requests for technical assistance for adult immunization schedule syndication can be sent to ncirdwebteam@cdc.gov.

## References

[1] Grohskopf LA, Sokolow LZ, Broder KR, Prevention and control of seasonal influenza with vaccines. MMWR Recomm Rep 2016;65(No. RR-5). 10.15585/mmwr.rr6505a1 27560619

[2] Meites E, Kempe A, Markowitz LE. Use of 2-dose schedule for human papillomavirus vaccination—updated recommendations of the Advisory Committee on Immunization Practices. MMWR Morb Mortal Wkly Rep 2016;65:1405–8. 10.15585/mmwr.mm6549a5 27977643

[3] Updated 2016 ACIP statement on October 2016 hepatitis B vaccination recommendations (publication pending).

[4] MacNeil JR, Rubin LG, Patton M, Ortega-Sanchez IR, Martin SW. Recommendations for use of meningococcal conjugate vaccines in HIV-infected persons—Advisory Committee on Immunization Practices, 2016. MMWR Morb Mortal Wkly Rep 2016;65:1189–94. 10.15585/mmwr.mm6543a3 27811836

[5] Updated ACIP statement on October 2016 meningococcal vaccination recommendations (publication pending).

[6] Kim DK, Riley LE, Harriman KH, Hunter P, Bridges CB. Advisory Committee on Immunization Practices. Recommended immunization schedule for adults aged 19 years or older, United States, 2017. Ann Intern Med 2017;166:209–18. http://annals.org/aim/article/doi/10.7326/M16-2936 26829913 10.7326/M15-3005

