# *Erratum*: Vol. 66, No. 5

**DOI:** 10.15585/mmwr.mm6608a8

**Published:** 2017-03-03

In the report “Advisory Committee on Immunization Practices Recommended Immunization Schedule for Adults Aged 19 Years or Older — United States, 2017,” on page 138, the fifth bullet point under the heading “Meningococcal vaccination” should have read “Young adults aged 16 through 23 years (preferred age range is 16 through 18 years) who are healthy and not at increased risk for serogroup B meningococcal disease may receive **either a 2-dose series of MenB-4C at least 1 month apart or a 2-dose series of MenB-FHbp at 0 and 6 months** for short-term protection against most strains of serogroup B meningococcal disease.”

